# Radionuclide, magnetic resonance and computed tomography imaging in European round back slugs *(Arionidae)* and leopard slugs *(Limacidae)*

**DOI:** 10.1038/s41598-021-93012-2

**Published:** 2021-07-05

**Authors:** Nicola Beindorff, Fabian Schmitz-Peiffer, Daniel Messroghli, Winfried Brenner, Janet F. Eary

**Affiliations:** 1 Berlin Experimental Radionuclide Imaging Center (BERIC), Charité - Universitätsmedizin Berlin, Berlin, Germany; 2 Department of Nuclear Medicine, Charité - Universitätsmedizin Berlin, Augustenburger Platz 1, 13353 Berlin, Germany; 3 Department of Internal Medicine - Cardiology, Deutsches Herzzentrum Berlin, Berlin, Germany; 4 Preclinical MRI Center, Charité - Universitätsmedizin Berlin, Berlin, Germany; 5 US National Institutes of Health/NCI/DCTD, Bethesda, USA

**Keywords:** Molecular biology, Zoology, Molecular medicine

## Abstract

Other than in animal models of human disease, little functional imaging has been performed in most of the animal world. The aim of this study was to explore the functional anatomy of the European round back slug (*Arionidae*) and leopard slug (*Limacidae)* and to establish an imaging protocol for comparative species study. Radionuclide images with single photon emission computed tomography (SPECT) and positron emission tomography (PET) were obtained after injections of standard clinical radiopharmaceuticals ^99m^technetium dicarboxypropane diphosphonate (bone scintigraphy), ^99m^technetium mercaptoacetyltriglycine (kidney function), ^99m^technetium diethylenetriaminepentaacetic acid (kidney function), ^99m^technetium pertechnetate (mediated by the sodium-iodide symporter), ^99m^technetium sestamibi (cardiac scintigraphy) or ^18^F-fluoro-deoxyglucose (glucose metabolism) in combination with magnetic resonance imaging (MRI) and computed tomography (CT) for uptake anatomic definition. Images were compared with anatomic drawings for the *Arionidae* species. Additionally, organ uptake data was determined for a description of slug functional anatomy in comparison to human tracer biodistribution patterns identifying the heart, the open circulatory anatomy, calcified shell remnant, renal structure (nephridium), liver (digestive gland) and intestine. The results show the detailed functional anatomy of *Arionidae* and *Limacidae*, and describe an in vivo whole-body imaging procedure for invertebrate species.

## Introduction

Much has been learned about comparative anatomy and physiologic systems from whole animal imaging in mammalian species and paleontology, however, little has been published that elucidates this information in live invertebrates^1,2^. In addition to providing detailed information on species morphometry for taxonomy investigations^3,4^, new insights might be gained in these unique models of environmental adaptation and function. These findings might apply to understanding effects of modern environments on ancient species and sustainability indicators in local populations. These findings however might also help define common areas in mammalian and invertebrate physiology as basis for comparative research, fostering in vivo experimental studies in invertebrates in general, and introducing molluscs as potential substitute for mammalian species in biomedical research.

The phylum mollusca is an invertebrate group comprised of gastropods (snails and slugs), cephalopods (squids and octopuses) and bivalves (clams and oysters). Common garden slugs or round back slugs and leopard slugs are terrestrial pulmonate gastropod mollusca. Terrestrial pulmonate slugs have a centralized nervous system, with one common neural ring around the gullet (esophagus), called the buccal mass. The two chamber heart, consists of one ventricle and one atrium connecting to an open circulation with major blood vessels leading from the lung to the heart, and aorta. Excretion takes place in the nephridium^5^.

Positron emission computed tomography (PET) in molluscs has been performed in *Octopus vulgaris* with ^18^F-fluoro-deoxyglucose (^18^F-FDG)^6^, and fluid transport in *Chironomus plumosus* larvae in muddy sediments has been imaged with ^18^F-fluoride in water^7^. Recently, PET biodistribution of ^18^F-FDG in honeybees has been reported^8^. Renal scintigraphy involving ^99m^technetium mercaptoacetyltriglycine (^99m^Tc-MAG3) and ^99m^technetium diethylenetriaminepentaacetic acid (^99m^Tc-DTPA) was used for the determination of kidney morphology and function in corn snakes (*Elaphe guttata guttata*) and green iguanas (*Iguana iguana*)^9,10^.

This imaging study was undertaken to (1) determine the feasibility of radionuclide imaging in invertebrates, the European round back slug (*Arion vulgaris, Arionidae*) and leopard slug *(Limax maximus, Limacidae)*, and (2) to determine if slug anatomic areas show differences in physiologic and metabolic activity measured by common radionuclide imaging procedures for major organ systems. For depicting anatomy, magnetic resonance imaging (MRI) and computed tomography (CT) were performed.

## Results

**Figure 1:**
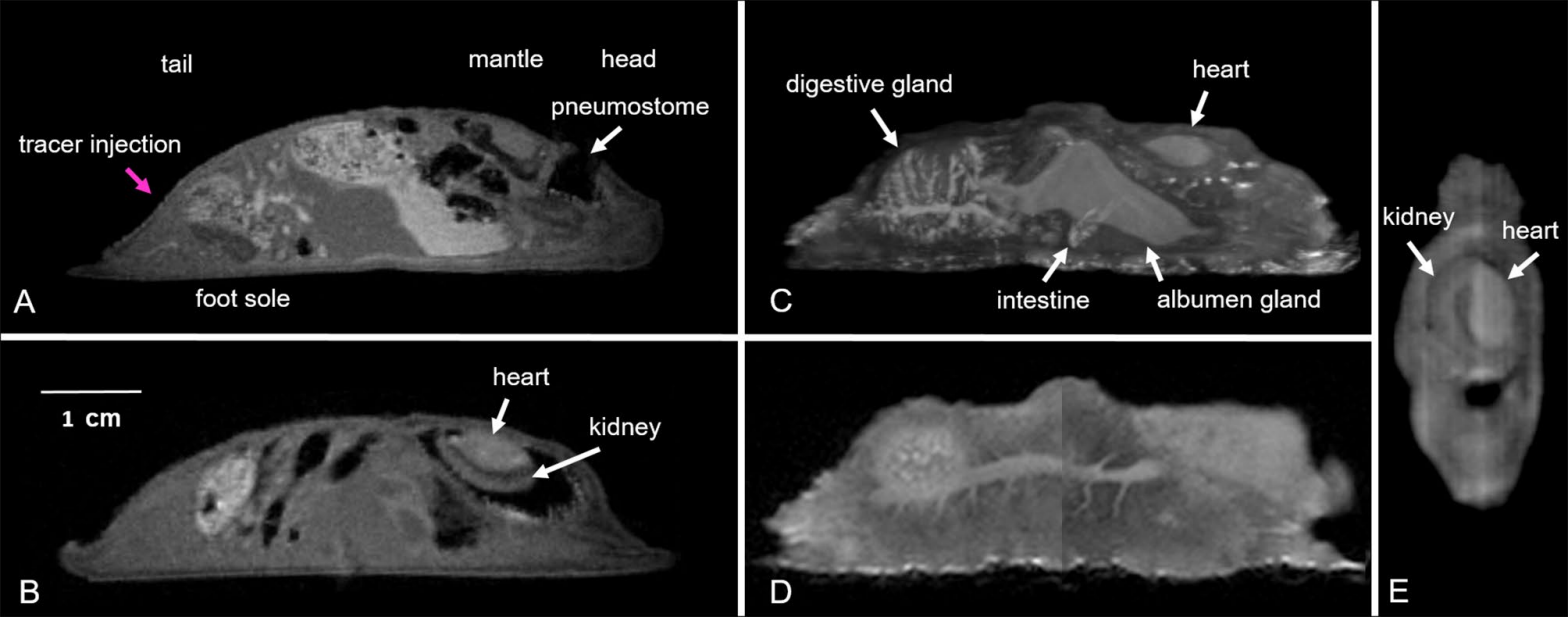
European round back slug (*Arionidae*); Sagittal 1 Tesla MR images (T1 GRE 3D, **A**, **B**): Pink arrow shows location of radiotracer tail injection. 3 Tesla MR images (T1 FSE 2D) with a lateral view maximum intensity projection (**C**). Sagittal image composed of two consecutive slices to illustrate the aorta with its branches (**D**), coronal image of the kidney and heart (**E**).

Soft tissue anatomy with MRI provided sufficient image quality for organ identification (Fig. 1A–E). The heart and kidney were identified directly beneath the mantle in the vicinity of various glands (Fig. 1B,C). Heart and vessel anatomy was visualized. The circulatory pathway of haemolymph passes from the heart to large arteries which branch to form increasingly smaller arteries as shown in Fig. 1D.

**Figure 2:**
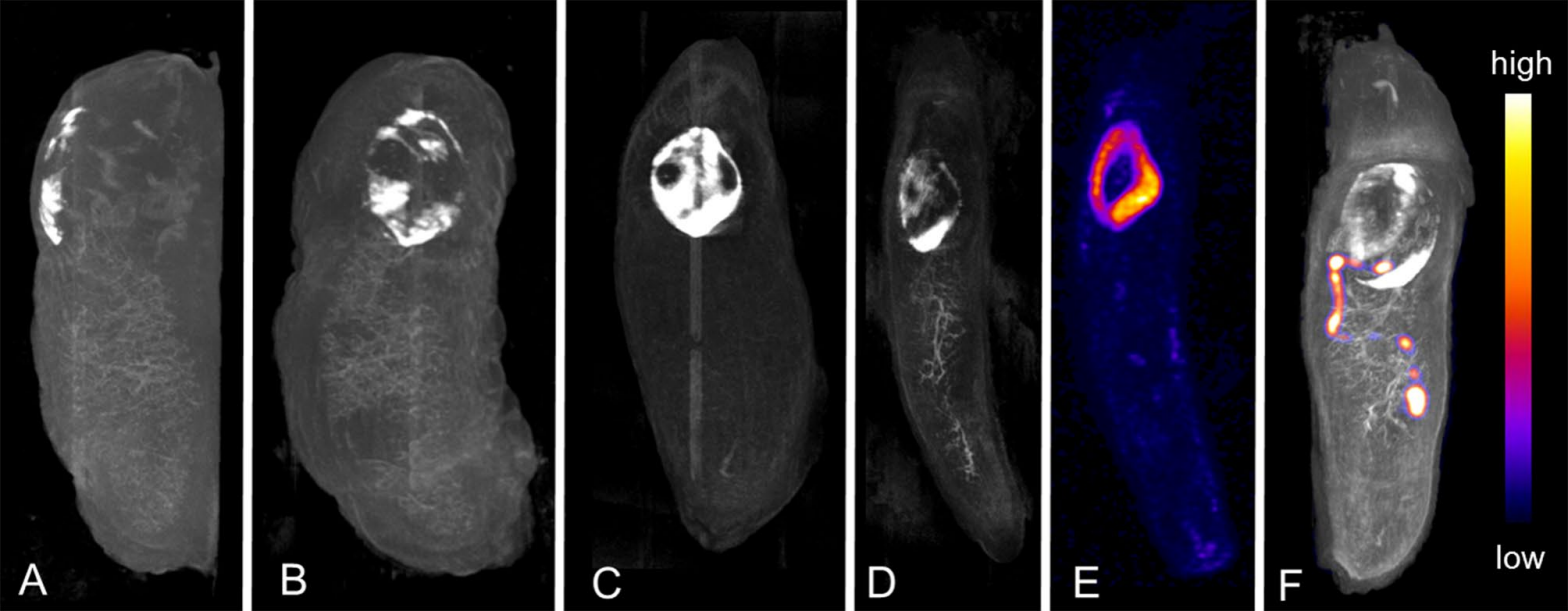
European round back slug (*Arionidae*); Maximum intensity projections of different animals showing their individual calcareous shell remnant pattern. CT images in lateral (**A**) and dorsal views (**B**–**D**, **F**). SPECT images after injection of ^99m^Tc-DPD with uptake in the kidney (**E**, dorsal view) and intestine (**F**, fused with CT). CT (**D**) and SPECT (**E**) images belong to a single individual with no ^99m^Tc-DPD shell remnant uptake.

The remnants of the reduced calcareous shell covered by the slug mantle collar is shown in detail in the CT image (Fig. 2A–D,F). Each slug shell is highly individual, similar to the human fingerprint.

Due to the open circulation system in slugs, there was rapid tracer distribution after tail injection. No residual tracer at tail injection locations was observed in images. Therefore, tail injection in a slug corresponds to intravenous injection in mammals distributing the tracer within the whole body with concentration in the respective specific target organs. For all tracers, the uptake for individual organs is shown as standardized uptake value from the 10 voxels with the highest uptake (SUVmax-10) in Table 1.

**Table 1:** Injected activity (MBq), time after injection, acquisition time and organ specific standardized uptake value from the 10 voxels with highest uptake (SUVmax-10) for each individual tracer.

Arionidae (n)	Limacidae (n)	Tracer	Injected mean activity (MBq)	Time after injection (min)	Acquisition time (min)	Figure	Organ SUVmax-10 (min–max)			
4	0	^99m^Tc-DPD	40	85–164	15	2E-F	Shell remnant 0	Kidney 17.1		
3	3	^99m^Tc-MAG3	19	27–50	15	3A-C	Kidney 59.5–114.1			
0	3	^99m^Tc-MAG3	19	dynamic 1–390	390	4A-B	Kidney 75 min 75.3–146.0	Kidney 2.6 h 67.0–190.1	Kidney 5 h 84.8–212.7	Kidney 6.5 h 208.8
0	3	^99m^Tc-DTPA	21	dynamic 1–430	430	4A, 4C	Kidney 75 min 30.8–50.6	Kidney 3 h 47.3–69.4	Kidney 5 h 50.8–70.7	Kidney 7 h 54.2–71.4
2	1	^99m^Tc-MIBI	56	70–153	15	3D-E	Heart 2.3–7.3	Kidney 2.6	Digestive gland 5.7–17.4	
2	2	^99m^TcO_4_^-^	77	26–85	13	3F-H	Kidney 3.4–32.7	Crop 1.1–4.4	Albumen gland 1.1–4.8	
2	2	^18^F-FDG	25	48–263	20	3I-J	Heart/kidney 0.9–4.7	Buccal mass 0.5–3.1	Intestine 1.8–2.3	Foot sole 1.1–2.6

^99m^Technetium dicarboxypropane diphosphonate (^99m^Tc-DPD) injection shows no uptake in the partially calcified shell remnant (Fig. 2). Because the kidney lies directly beneath the shell remnant, kidney ^99m^Tc-DPD (10.3%IA, 1.7 h post injection) excretion could be mistaken for shell remnant uptake. This finding is demonstrated in Fig. 2D,E. In the same slug, the shell remnant (D) and kidney (E) have a similar shape. Interestingly, the kidney SUVmax-10 in this slug is 17.1 after 102 min, while kidney SUVmax-10 of the kidney specific tracers ^99m^Tc-MAG3 and ^99m^Tc-DTPA are in the range of 75.3–146.0 and 30.8–50.6 after 75 min, respectively. In a different animal shown in Fig. 2F, ^99m^Tc-DPD uptake is in the intestine and no shell uptake is seen.

**Figure 3:**
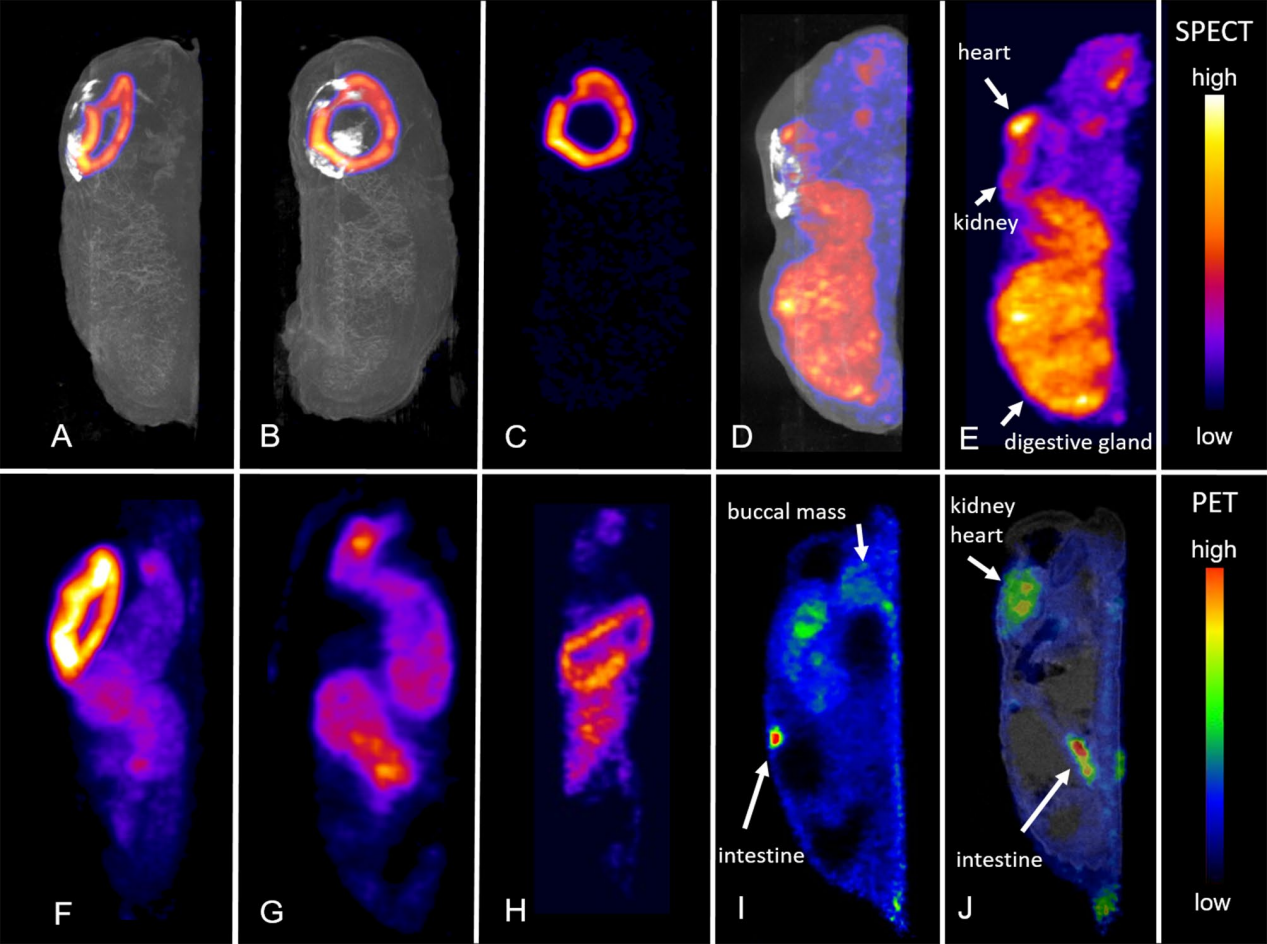
European round back slug (*Arionidae*); Radionuclide images. SPECT images after tracer injection (**A**–**H**, maximum intensity projections): ^99m^Tc-MAG3 uptake in the kidney in lateral (**A**) and dorsal view (**B**) fused with CT, and SPECT image alone (**C**). ^99m^Tc-MIBI uptake images in lateral views fused with CT (**D**) and alone (**E**). ^99m^Tc-MIBI uptake foci are located in the heart, kidney and digestive gland. SPECT images showing ^99m^TcO_4_^−^ uptake in the kidney (**F**, lateral view), crop and albumen gland in coronal projections (**G**, **H**). Sagittal ^18^F-FDG PET image alone (**I**) and fused with MRI (**J**).

**Figure 4:**
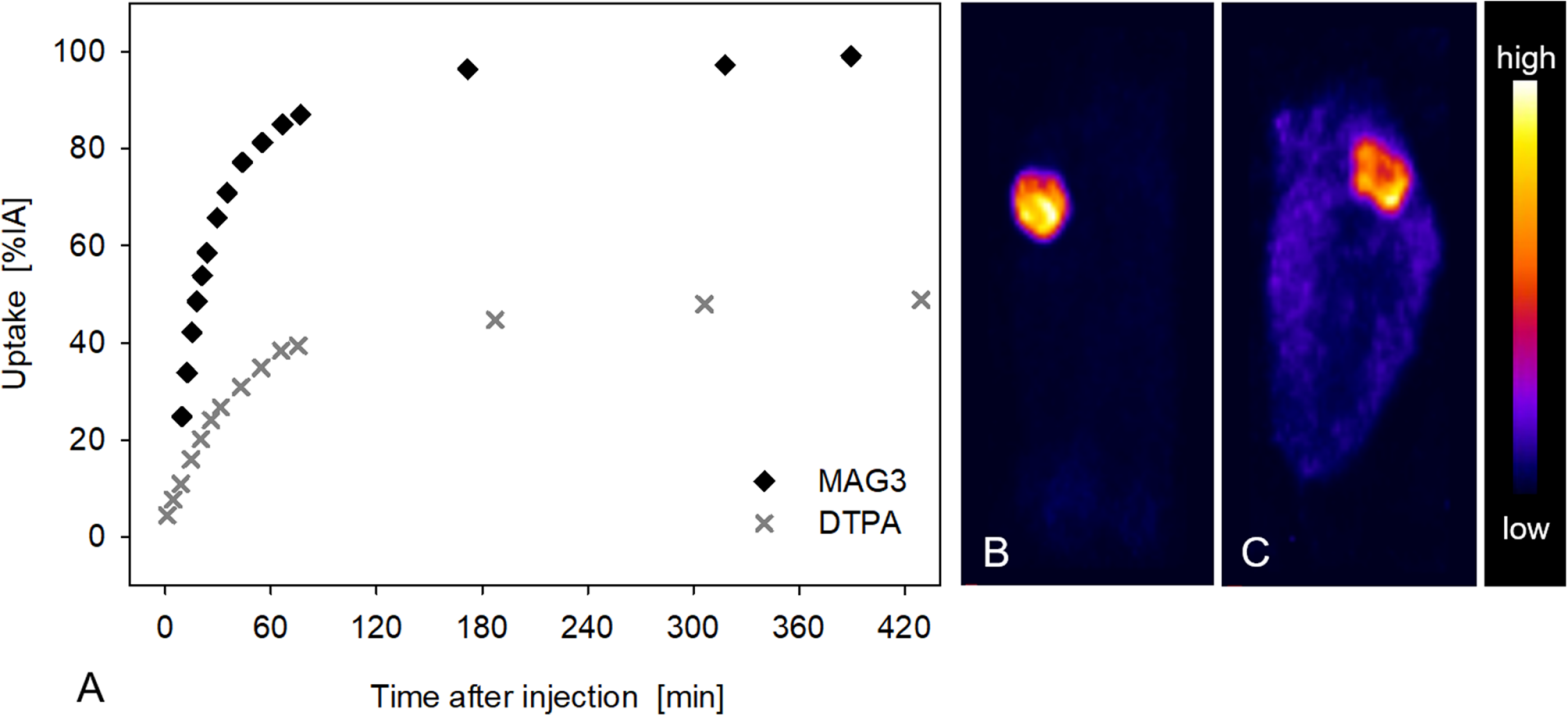
Kidney time activity curves (TAC) from dynamic SPECT initiated at the time of injection of ^99m^Tc-MAG3 and ^99m^Tc-DTPA in a leopard slug (*Limacidae*) each (**A**). SPECT images with kidney uptake of ^99m^Tc-MAG3 (**B**) and ^99m^Tc-DTPA (**C**) 15 min after tracer injection (maximum intensity projections, lateral-dorsal view).

^99m^Technetium sestamibi (^99m^Tc-MIBI) images show intense uptake in the digestive gland after 1.2 h (Fig. 3D,E). Kidney excretion occurs, and additional uptake is identified in the heart (0.5%IA).

Figure 3A–C shows ^99m^Tc-MAG3 kidney uptake 30 min after injection. The nephridium (kidney) is clearly seen. Whole animal single photon emission computed tomography (SPECT) images demonstrate complete tracer distribution and concentration only in the kidney after tail injection. ^99m^Technetium diethylenetriaminepentaacetic acid (^99m^Tc-DTPA) kidney uptake images correspond to ^99m^Tc-MAG3 uptake. Kidney ^99m^Tc-MAG3 and ^99m^Tc-DTPA uptake kinetics are shown in Fig. 4A with typical renal time activity curves (TAC) in leopard slugs. TAC for the kidneys were highly congruent in all animals. Renal TAC showed a steep increase after both ^99m^Tc-MAG3 and ^99m^Tc-DTPA injection. Maximum kidney uptake of nearly 100%IA ^99m^Tc-MAG3 and 45%IA ^99m^Tc-DTPA was reached between 40 and 60 min after tracer injection. Once maximum kidney uptake was reached, there was no decline in uptake during the subsequent imaging period up to 7 h. As early as 15 min after tracer injection, rapid kidney ^99m^Tc-MAG3 uptake is evident in Fig. 4B. In the entire animal, uptake is almost exclusively in the kidney with very small background activity. In contrast, for ^99m^Tc-DTPA, the slower and lower uptake into the kidney clearly shows the higher tracer content in the whole slug at 15 min (Fig. 4C). The higher tracer uptake in the kidney for ^99m^Tc-MAG3 than for ^99m^Tc-DTPA is also confirmed by a higher SUVmax-10 as shown in Table 1.

Figure 3F–H shows ^99m^technetium pertechnetate (^99m^TcO_4_^−^) imaging with kidney excretion (Fig. 3F), uptake in the crop and stomach (Fig. 3F–G) located in the organ wall (Fig. 3H).

^18^F-FDG PET images were successfully obtained (shown in Fig. 3I,J). The highest ^18^F-FDG uptake was identified in the heart and kidney, intestine, buccal mass, albumen gland and foot sole. Uptake was markedly lower in the remaining organs.

## Discussion

This study demonstrates the first use of nuclear medicine techniques for live, non-invasive whole animal imaging of physiologic radiotracer uptake in combination with MRI and CT in round back *(Arionidae)* and leopard slugs *(Limacidae)*. By administering six different standard tissue-specific radionuclide tracers commonly applied for functional and metabolic nuclear medicine studies in humans, major organs such as kidney and heart in slugs were imaged and physiological tracer uptake was measured.

In life animals, motion artefacts are a major obstacle for both high-quality and quantitative images. To avoid motion of the slugs during imaging, hypothermia was performed. After the imaging study, animals resumed movement as soon as they warmed to room temperature. Consequently, hypothermia proved to be a suitable method for immobilization during imaging, effectively reducing motion artefacts. As roundback and leopard slugs are pulmonate slugs, immobilization by inhalational anesthesia with isoflurane seemed feasible as well. In contrast to hypothermia, this method however, caused the animals to steadily elongate over a long period of time by relaxing, thus causing increasingly motion artefacts during imaging. Furthermore, the animals needed several hours to wake up and recover. Inhalation anesthesia, therefore, proved to be less suitable for imaging studies.

^99m^Tc-MAG3 used for kidney functional imaging in humans is taken up by kidney tubular cells with a high extraction fraction of 40–50% at each passage through the kidney and subsequently excreted into the urine^11^ allowing calculation of tubular excretion rate. As shown in Fig. 3A–C ^99m^Tc-MAG3 is also almost completely taken up by the slug nephridium with no other areas of significant uptake. This finding indicates that tubular transport mechanisms in slug kidneys are similar to mammalian kidneys. ^99m^Tc-DTPA, a tracer for quantitative determination of the kidney glomerular filtration rate, is also taken up by the slug kidney but approx. 50% less than ^99m^Tc-MAG3 (Fig. 4A). Similarly, in humans the ^99m^Tc-MAG3 tubular excretion rate is higher than the ^99m^Tc-DTPA glomerular filtration rate resulting in a higher peak uptake in the kidney^12^. The metanephridium with tubular cells is found in many molluscs. On the other hand, glomeruli in kidneys of molluscs are not reported in literature. Interestingly, still approx. 45% of the injected activity of ^99m^Tc-DTPA is concentrated in the slug kidney. While water gastropods excrete a highly diluted primary urine with ammonia, in terrestrial gastropods reabsorption of water takes place to a greater extent^13^. In terrestrial pulmonate gastropods, excretion generally occurs in form of uric acid, which contains very little water and crystallizes into whitish crystals on the outer wall of the kidney septa^14,15^. This slow renal excretion mechanism is reflected by the uptake plateau in the TAC up to 7 h after injection (Fig. 4A) and does not correspond to mammalian kidney tracer excretion and human kidney TAC which reaches the maximum uptake within minutes and then continuously decreases due to excretion into the urine^16^.

In humans, ^99m^Tc-MIBI is taken up by viable cardiomyocytes according to blood flow and transported into mitochondria where it binds to the organelle inner membrane through an electronegative transmembrane potential gradient^17^. This tracer is primarily excreted by the liver into the intestine and to a much lesser extent by the kidneys. Even if the specific uptake into mitochondria of the slugs could not be proven, these results indicate that the same organs are visualized in slugs as in humans where images depict tracer uptake in the heart, nephridium, and mainly in the digestive gland (liver equivalent) in *Arionidae* (Fig. 3D,E).

In slugs, kidney ^99m^Tc-DPD excretion is observed (Fig. 2E) similar to human excretion while the calcareous shell remnant, easily seen on CT images (Fig. 2A–D,F), did not show ^99m^Tc-DPD uptake. This finding is most likely based on differences in shell chemical composition and mineral structure compared to the vertebrate skeleton. DPD is a phosphate-based mineral that binds to hydroxyapatite in the mammalian skeleton. The slug shell remnant is composed of calcite or aragonite, and other orthorhombic carbonate minerals^18^.

^18^F-FDG metabolic activity has a biodistribution pattern in slugs similar to humans with characteristic uptake in heart, liver, and active muscle. Areas of uptake include the intestine, and kidney excretion. In contrast to the high glucose uptake in the human brain, the cerebral ganglion in round back and leopard slugs is either too small for system image resolution or the metabolism level is too low for uptake to be distinguished from the buccal mass.

^99m^TcO_4_^−^ is a substrate for the sodium-iodide symporter (NIS), a transporter expressed in thyroid, salivary and stomach parietal cells^19,20^. As inorganic anion, it undergoes kidney excretion. In slugs, ^99m^TcO_4_^−^ uptake was identified in the nephridium, but also in the crop and digestive glands. This ^99m^TcO_4_^−^ uptake pattern may be specific although we were not able to prove NIS expression by immunostaining with human or mouse anti-NIS antibodies (data not shown). It has been shown however, that thyroxine (T_4_; tetra-iodo-thyronine) has existed in invertebrates of the Phylum Porifera (sponges) and Anthozoa (sea anemones, corals) for 700 million years^21^.

The findings summarized above might help define common areas in mammalian and invertebrate physiology as basis for comparative and phylogenetic research, and fostering in vivo experimental studies in invertebrates. Furthermore, in vivo functional and morphologic imaging can be used to determine the anatomy of a species without sacrificing the animals.

Furthermore, these studies introduce molluscs as potential substitute for mammalian species in biomedical research analogous to other non-mammalian model systems such as the chicken egg chorion-allantois membrane assay^22^. In literature reports, the Slug Mucosal Irritation test to assess the local tolerance of pharmaceutical products applied to the mucosa has been successfully used in both *Arionidae* and *Limacidae*, thus, allowing to substitute the use of vertebrates (e.g. rabbits) as test organisms^23^.

Our findings might also support efforts to understand the effects of modern environments on species and sustainability indicators in local populations. Terrestrial molluscs species exist by consuming plant materials and are a food source for birds and other animals. They may be sources of concentrated man made soil and vegetation toxins transmitted to other animals in the food chain. In snails (*Achatina fulica*), ingested microplastic fibers were shown to reduce food intake and excretion. Significant villi damage occurred in the gastrointestinal walls but did not affect liver and kidney histology^24^. The present study indicates that kidneys of molluscs in particular can be imaged non-invasively by in vivo functional and quantitative scintigraphy. In snakes and iguanas, renal scintigraphy has been already successfully performed and suggested as a useful tool for the diagnosis of renal failure in reptiles^9,10^.

The advantage of imaging is the ability to examine animals and specific organ functions in vivo multiple times during an observation period. Consequently, successive studies under environmental conditions with pollution or reversible influences after the removal of noxious agents can be studied in one and the same animal. Thus, the ability to collect longitudinal quantitative radionuclide data on organ function in molluscs may foster environmental and toxicological in vivo studies in ecosystem research.

## Methods

**Figure 5:**
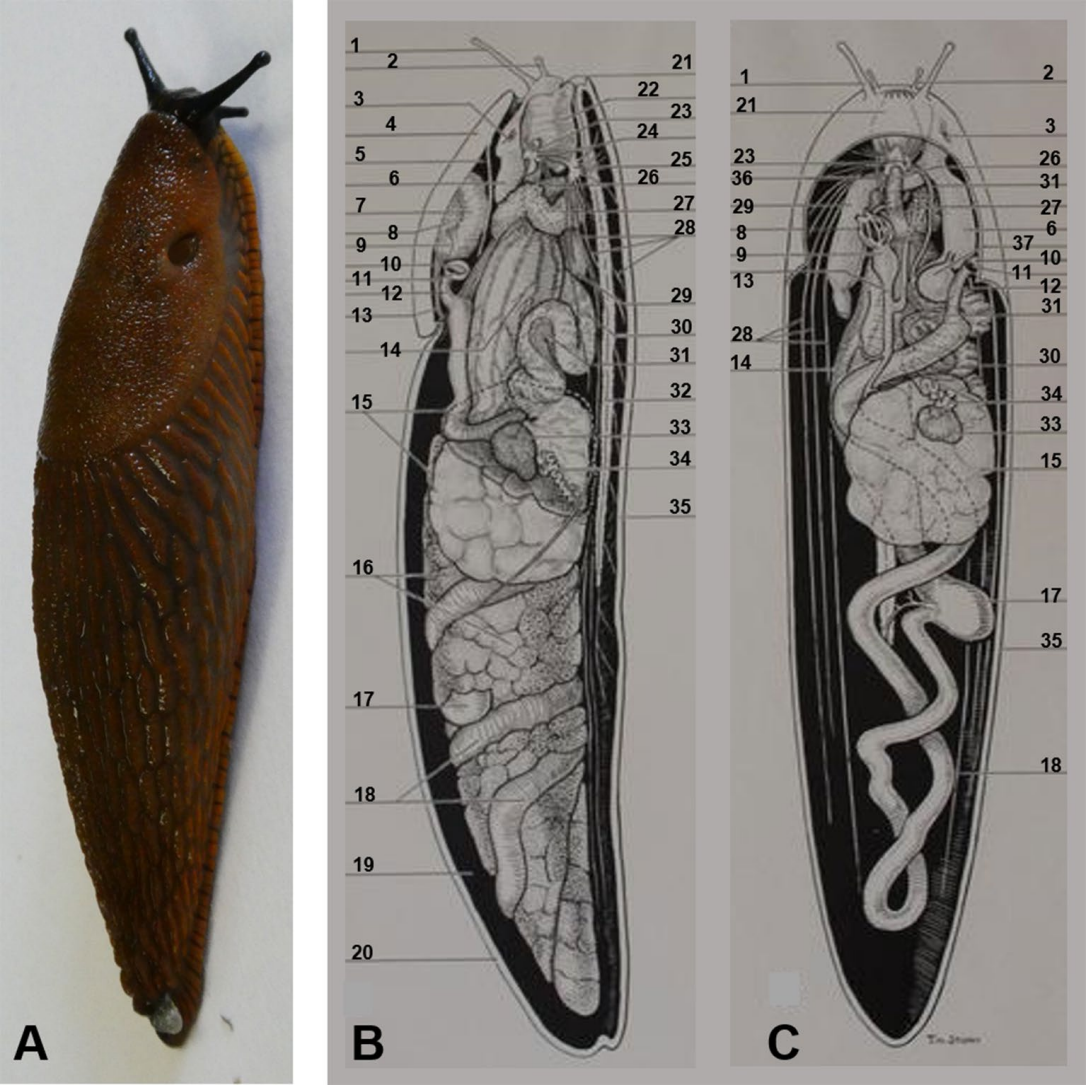
(**A**) European round back slug (*Arionidae*). (**B**, **C**): *Arionidae* anatomy drawing adapted from classroom chart Copyright 1952 by General Biological Supply House, 8200 South Hoyne Avenue, Chicago, Illinois 60,620, printed in U.S.A. (**B**) cross section, viewed from right side, (**C**) cross section, viewed from above, 1 tentacle with eye at end, 2 tentacle, 3 genital aperture, 4 shield (part of mantle over pulmonary space), 5 pulmonary space, 6 vagina, 7 kidney (nephridium), 8 auricle of heart, 9 ventricle of heart, 10 pulmonary opening, 11 anus, 12 rectum, 13 aorta, 14 crop, 15 albumen gland, 16 digestive gland or (liver), 17 gastric caecum, 18 intestine, 19 mantle cavity, 20 mantle, 21 mouth, 22 buccal mass containing the radula and cartilages, 23 cerebral ganglion, 24 connective nerves, 25 subesophageal ganglion, 26 esophagus, 27 salivary gland, 28 nerves, 29 penis, 30 oviduct, 31 sperm duct, 32 mucus gland or slime gland, 33 ovotestis, 34 hermaphroditic duct, 35 foot, 36 salivary duct, 37 spermatheca.

European round back slugs (*Arion vulgaris, Arionidae*, Fig. 5A) and leopard slugs (*Limax maximus, Limacidae)* were collected in Germany from a domestic garden. For SPECT, PET, MRI and CT slugs were kept in a 2 L transparent plastic box with a top air filter at room temperature (22 °C), and fed with salad greens and apples ad libitum. Humidity in the cage environment was maintained with wet bedding fashioned from assorted garden plants. The radiopharmaceuticals used were ^99m^technetium dicarboxypropane diphosphonate (^99m^Tc-DPD), ^99m^technetium mercaptoacetyltriglycine (^99m^Tc-MAG3), ^99m^technetium diethylenetriaminepentaacetic acid (^99m^Tc-DTPA), ^99m^technetium pertechnetate (^99m^TcO_4_^−^), ^99m^technetium sestamibi (^99m^Tc-MIBI) and ^18^F-fluoro-deoxyglucose (^18^F-FDG).

Thirteen round back slugs (body weight 5–13 g) and 11 leopard slugs (body weight 2–14 g) were injected with 0.1 ml tracer into the tail (Fig. 1A) using a 30G sterile syringe for a total of 27 scintigraphic studies. The lack of accessible blood vessels for intravenous tracer administration necessitated tail injection. The open circulation system in slugs leads to rapid tracer distribution similar to intravenous injections in mammalian species. After radiotracer injection, slugs were placed separately in a 1 L glass jar with salad greens at room temperature. Table 1 shows the number of examinations, injected activity (MBq) and incubation time for each radiotracer uptake. To prevent motion during imaging, the slugs were placed in a 50 ml centrifuge tube in a − 20 °C freezer immediately prior to imaging for a maximum of 10 min to prevent ice crystal formation and frostbite. After a maximum imaging time of 30 min, slugs were put back into the plastic box. They resumed movement as soon as they warmed to room temperature, about 20 min.

SPECT and CT imaging were performed using the NanoSPECT/CTplus scanner (Mediso, Hungary /Bioscan, France). The dual modality system for dedicated small animal imaging consists of a quadruple head gamma camera with NaI(TI) crystal detectors. Each detector is equipped with a nine-pinhole aperture (mouse high resolution, d = 1.0 mm) with a spatial resolution of 0.7 mm FWHM for ^99m^technetium^25^. SPECT images were acquired for 10–20 min using 20 projections consisting of five steps displaced by 18° and the energy window was set at 140 keV ± 10%. CT images were performed with the following parameters: matrix 232 × 232 × 470 with dimensions 0.15 × 0.15 × 0.15 mm^3^, 45 kVp, 500 ms exposure time and 360 projections.

To study renal uptake kinetics, six leopard slugs were imaged at room temperature. The slugs were injected with ^99m^Tc-MAG3 or ^99m^Tc-DTPA (n = 3 each) for kidney uptake kinetics directly before start of the SPECT acquisition. The whole slug was put in a shortened 20 ml syringe with a width of 22 mm for motion restriction and was placed transversely within the 22 mm field of view for semi-stationary SPECT. Semi-stationary dynamic SPECT acquisition with intersampling was performed with two angular steps per time frame displaced by 45° as described by Huang et al.^16^. The dynamic data set consisted of time frames with 10** × **20 s (10 s per detector position) followed by 60** × **50 s frames (25 s per detector position). Thereafter, the slugs were imaged at 3 further time points with 5** × **50 s frames up to 7 h. The short time frames allowed to follow the kidney for image analysis during slug potential movement thus enabling correction for movement.

PET/MR imaging was performed using a dedicated small animal 1 Tesla nanoScan PET/MRI (Mediso, Hungary). MRI scans were acquired using a T1-weighted 3D spoiled gradient echo sequence (T1 GRE 3D) with the following parameters: coronal and sagittal sequential acquisitions, matrix 256** × **256** × **58 with dimensions 0.23** × **0.23** × **0.5 mm^3^, TR: 15 ms, TE: 2.9 ms, and flip angle 25°. An additional T1-weighted GRE for attenuation correction of the PET-image was conducted with the following parameters: matrix 144** × **144** × **163 with dimensions 0.5** × **0.5** × **0.6 mm, TR: 25 ms, TE: 2.0 ms, and flip angle 30°. PET imaging was performed for 20 min (matrix 97** × **101** × **315, dimensions 0.3** × **0.3** × **0.3 mm^3^). Further MR images in one slug were obtained with a small animal 3Tesla MRI system (MRS 3017, MR Solutions, United Kingdom) using a T1-weighted fast spin echo sequence (FSE multi-slice 2D) with the following parameters: sagittal sequential acquisitions, matrix 256** × **256** × **48 with dimensions 0.2** × **0.2** × **1.1 mm, TR: 720 ms, TE: 11 ms, averages: 4–5.

Tracer uptake in the slug organs was determined by manual contouring of volumes of interest (VOI) in the SPECT and PET images applying CT and/or MR images for exact organ localization using PMOD 3.5 (PMOD Technologies Ltd., Switzerland). A standardized uptake value was computed from the 10 voxels showing the highest uptake activity within the tracer-positive organs (SUVmax-10). Additionally, the percentage tracer uptake in the organs was calculated by normalizing the integrated activity of the VOI to the total injected activity (%IA).

Detailed anatomy of the slug is illustrated in Fig. 5B,C.
